# The paternal brain: longitudinal insights into structural and functional plasticity and attachment over 24 weeks postpartum

**DOI:** 10.1038/s41398-026-04082-7

**Published:** 2026-05-14

**Authors:** Negin Daneshnia, Elena Maria Losse, Angela Kurz, Natalia Chechko, Susanne Nehls

**Affiliations:** 1 Department of Psychiatry, Psychotherapy and Psychosomatics, Faculty of Medicine, RWTH Aachen, Aachen, Germany; 2 Institute of Neuroscience and Medicine: JARA- Institute Brain Structure Function Relationship (INM 10), Research Center Jülich, Jülich, Germany; 3 Institute of Neuroscience and Medicine, Brain & Behavior (INM-7), Research Center Jülich, Jülich, Germany

**Keywords:** Neuroscience, Biological sciences

## Abstract

Over the last two decades, interest in the neurobiological basis of parenthood has grown, with the primary focus being on maternal neuroplasticity. However, research on paternal brain adaptations remains limited and inconclusive. This longitudinal study investigated gray matter volume (GMV) and resting-state functional connectivity (rsFC) changes in 25 fathers immediately after childbirth and at 3, 6, 9, 12, and 24 weeks postpartum. Morphological changes were most evident as reductions in GMV within the bilateral occipital, frontal, temporal and parietal cortices, as well as the temporo-parietal junction, and the insular and hippocampal regions during the first six weeks, followed by gradual stabilization. From 12 weeks onward, local GMV increases were observed in the frontal and cerebellar regions. Connectivity analysis revealed significant reorganization within the Salience, Default Mode, and Frontoparietal Networks, with peak changes during the first 9 weeks marked by a shift from sensory processing to enhanced cognitive and affective processing. Additional rsFC analysis identified increased amygdala-cingulate and amygdala-hippocampal connectivity, indicating a significant link between the amygdala and paternal attachment during the first 12 weeks. These findings outline a clear trajectory of paternal neuroplasticity and adaptation during the early postpartum period, followed by maintenance and fine-tuning processes that likely facilitate paternal caregiving behaviors. **Clinical Trial Registration:** The study was registered at the German Clinical Trials register (DRKS; ID: DRKS00024875).

## Introduction

The transition to parenthood marks one of the most transformative periods in human life, requiring new parents to adapt to a new life-long role. It is also considered to be a period of pronounced neural plasticity [1, 2]. With its traditional focus on women, research has accumulated a large body of evidence regarding the restructuring of the maternal brain, which is thought to be triggered by co-varying hormonal changes [3, 4]. Collectively, these studies suggest a pregnancy-related reduction in gray matter volume (GMV) and cortical thickness during gestation, followed by region-specific and time-sensitive changes in GMV and resting-state functional connectivity (rsFC) across the postpartum period [4, 5]. These alterations encompass widespread regions that comprise the so-called “maternal brain” [6] and have been linked to maternal caregiving behaviors, including motherly attachment [4, 7]. Notably, adaptations of the amygdala have been linked to lower hostility toward the child, highlighting experience-dependent neural changes [4]. Although (soon-to-be) fathers do not undergo the immense endocrinological and physiological changes as mothers, they do have to adapt to meet the new demands of fatherhood. Studies have gathered evidence of paternal endocrinological adaptations both before and after childbirth [8, 9], including lower testosterone and higher prolactin levels, compared to non-fathers [10, 11], which are believed to facilitate paternal attachment [8] and sensitivity [12, 13]. Moreover, elevated paternal cortisol levels during the postpartum period have been linked to infant executive functioning [14], highlighting the broader significance of these biological adaptations.

The critical role of fathers is being increasingly recognized, with research emphasizing the significant impact of paternal involvement on infant socioemotional and cognitive development [15–17]. While there have been a few studies investigating neuroplasticity changes related to paternity, the findings remain inconsistent, likely due to the studies’ designs, particularly the lack of standardized measurement time points. Kim et al. have reported increased GMV in subcortical structures (e.g., the hypothalamus, the amygdala, the putamen, the caudate) at 2–4 weeks and 12–16 weeks postpartum (*n* = 16), alongside reductions in the orbitofrontal cortex (OFC), the insula, the posterior cingulate cortex, the superior temporal and medial frontal gyri [18]. Other studies have measured fathers during their partners’ pregnancies and several months postpartum (2–9 months after childbirth). In contrast to previous findings, the results of these studies have identified postpartum cortical volume reductions across the entire cortex (*n* = 38) [19] and within the visual and default mode network (DMN; *n* = 40) [20], showing the subcortical structures to remain preserved [19, 20].

While the evidence of structural changes in the paternal brain remains inconclusive, functional neuroimaging studies have highlighted distinct brain activation patterns in fathers compared to non-fathers. Specifically, fathers have been found to exhibit altered activation in distinct cortical regions involved in socio-cognitive and emotional processing [13, 21–23], as well as in large-scale networks [24, 25]. These effects have been reported from as early as 2–4 months postpartum [13] to up to 3 years postpartum [22], with the fathers being assessed one or two times across studies (*n* = 10–21) [13, 21–23]. Moreover, region-specific functional connectivity has been shown to be directly linked to parental caregiving behaviors. For instance, increased paternal amygdala connectivity with regions involved in empathy and social cognition has been seen to be positively linked to greater involvement in childcare [26, 27]. Similarly, maternal studies have shown increased amygdala connectivity following childbirth, along with significant associations with caregiving [28], highlighting the amygdala’s central role in attachment and parental behaviors. At the network level, paternal connectivity patterns closely mirror those observed in the maternal postpartum brain [5, 29, 30], indicating that parenting-related neural adaptation may be universal rather than sex-specific. In support of this, Feldman has proposed a global “parental brain network” as an evolutionary adaptation [30] involving structures such as the empathy network (i.e. the anterior cingulate cortex, anterior insula, and supplementary motor area) for emotional attunement [31] and the emotion regulation network (i.e. the dorsolateral prefrontal cortex, medial orbitofrontal cortex, middle frontal gyrus, and frontopolar cortex) for affect regulation and multitasking during childrearing [32]. However, compared to maternal neuroplasticity, the neural processes underlying fatherhood remain poorly understood.

To address this gap, we conducted a longitudinal study over 24 weeks, collecting both anatomical and rsFC imaging data during this period. In light of the heterogeneity of study designs and the variability of findings in previous paternal neuroimaging research, we employed strict and relatively short measurement intervals to systematically capture both early and evolving neuroplastic changes. Following the methodology of our prior research on maternal postpartum brain adaptations [3–5], data were acquired starting within the first week postpartum, with assessments at three-week intervals until 12 weeks, and a final assessment at 24 weeks.

We hypothesized that fathers would show GMV changes in brain regions associated with the parental caregiving network, including areas involved in emotion regulation, mentalizing, and empathy [26, 30, 33]. In line with the findings on maternal postpartum neuroplasticity [3, 4], we expected these alterations to follow a temporal trajectory, emerging shortly after childbirth and continuing to develop over the 24-week period. Additionally, we anticipated dynamic alterations in intrinsic functional connectivity in paternal brains over time. Building on prior studies on paternal brain network connectivity [13, 21, 22, 34] and its overlap with maternal brain activity [5, 29, 30], we expected these changes to involve key brain networks including the Default Mode Network (DMN), the Salience Network (SN), and the Frontoparietal Network (FPN). Finally, we explored potential neural correlates of paternal attachment. Building on prior evidence linking maternal attachment to structural brain changes [4], as well as findings implicating amygdala connectivity in caregiving and attachment processes in both fathers [26, 27] and mothers [28], we conducted exploratory analyses examining the associations between paternal attachment and (1) GMV changes as well as (2) inter-regional functional connectivity of the amygdala as a candidate neural correlate. These analyses aimed to complement our broader investigations of GMV and large-scale network dynamics in the paternal postpartum brain.

## Methods

### Design and participants

The present research was evaluated and approved by the Ethics Review Board of the University Hospital Aachen and it complied with the ethical standards of the Helsinki declaration. During their partners’ stay in the Department of Gynecology and Obstetrics, University Hospital RWTH Aachen, biological fathers (*n* = 26) were recruited within 1–6 days of childbirth. Informed consent was obtained from all subjects prior to their participation in the study. For additional information on the recruitment procedure, please see SI.

MRI measurements took place within the first week of childbirth, as well as after 3, 6, 9, 12 and 24 weeks postpartum. For each session, the average measurement time frames were 4.96 (SD = 1.94) days postpartum for T0, 20.84 days (SD = 2.01) for T1, 41.6 days (SD = 2.78) for T2, 61.52 days (SD = 2.39), 80.44 days (SD = 2.88) for T4, and 168.32 days (SD = 13.44) for T5. At each MRI measurement time point, subjects were asked to complete the Paternal Postnatal Attachment Scale (PPAS) [35], an instrument assessing father-to-infant attachment. Derived scores included a total score of paternal attachment as well as three subscales for Quality of Attachment, Absence of Hostility, and Pleasure in Interaction. The data of one subject were excluded due to a severe illness during the study. All retained participants contributed complete datasets across all six time points. Thus, the data of 25 postpartum men were utilized for analyses (mean age = 32.6, SD = 4.09, range = 24–42). Based on an a priori power analysis, the study was designed to detect medium-to-large effect sizes. All details regarding sample size considerations and a priori power analysis are provided in the Supplementary Information. A summary of the characteristics of the sample is provided in Table 1.

**Table 1:** Socio-demographic information and paternal attachment of men in the postpartum period.

	n (%)	Mean ± SD
Paternal Age (years)		32.6 ± 4.09
Country of origin		
Germany	19 (76)	
Southwest Asia	4 (16)	
Other	2 (8)	
First-time father	22 (80)	
Partners’ gestational age in weeks		39.12 ± 1.83
Child’s birth weight (g)		3319.12 ± 599.75
Partners’ birth mode		
C-section	12 (48)	
Spontaneous	10 (40)	
Ventouse	3 (12)	
Secondary education		
Lowest (<9 years)	2 (8)	
Middle (10–12 years)	5 (20)	
Highest (>13 years)	18 (72)	
Paternal leave		
Yes	14 (56)	
No	11 (44)	
Time spent at home after childbirth (months)		2.36 ± 3.24
Household income		
20–50k	4 (16)	
> 50k	21 (84)	
Married (yes)	20 (80)	
PPAS 3 weeks pp		83.74 ± 7.23
Quality of attachment		41.87 ± 2.76
Absence of hostility		20.83 ± 3.77
Pleasure in interaction		21.04 ± 2.8
PPAS 6 weeks pp		83.69 ± 7. 22
Quality of attachment		41.56 ± 3.27
Absence of hostility		21.13 ± 2.97
Pleasure in interaction		21 ± 3.22
PPAS 9 weeks pp		82.04 ± 7.93
Quality of attachment		41.26 ± 3.16
Absence of hostility		20.30 ± 3.15
Pleasure in interaction		20.48 ± 4.02
PPAS 12 weeks pp		81.91 ± 9.49
Quality of attachment		41.30 ± 4.31
Absence of hostility		19.95 ± 2.98
Pleasure in interaction		20.65 ± 4.15
PPAS 24 weeks pp		82.74 ± 9.9
Quality of attachment		41.69 ± 4.38
Absence of hostility		20.48 ± 2.99
Pleasure in interaction		21.87 ± 2.76

PPAS [35], *pp* postpartum.

#### Anatomical and resting-state functional MRI data acquisition and processing

MRI acquisitions were performed on a 3 Tesla Prisma MR Scanner (Siemens Medical Systems, Erlangen, Germany) located in the Medical Faculty of University Hospital Aachen. Structural T1-weighted images were acquired using 3-dimensional magnetization-prepared rapid acquisition gradient echo imaging sequence, while whole-brain echoplanar images (EPIs) were acquired during rest with eyes closed. Anatomical MRI data were preprocessed using the Computational Anatomy Toolbox (CAT12.8.2 Version r2170) and statistical parametric mapping (SPM) 12 toolbox, implemented in Matlab 2019b (MathWorks). Images of each subject were preprocessed according to the longitudinal protocol in CAT12 [36]. For resting-state data, preprocessing was conducted utilizing the default preprocessing pipeline of CONN functional connectivity toolbox (CONN, version 22.a, https://www.nitrc.org/projects/conn) implemented in SPM12 and Matlab 2019b (MathWorks). Please see SI for a detailed overview of the protocol and preprocessing of structural and functional data.

##### Analyses of VBM

For the voxel-based morphometry (VBM) analyses, total cranial volume (TIV) was used as a covariate [36]. In line with CAT12 recommendations for longitudinal analyses, age was not included as a covariate. A flexible-factorial general linear model (GLM) was utilized, including the two factors subject and time point. Significant main effects were pursued with post-hoc t-contrasts. Additionally, we used multivariate regression analyses to examine associations between whole-brain GMV and paternal attachment (PPAS total and subscales), both at each time point and for GMV changes between time points. Full methodological details are provided in the SI. Unless specified otherwise, for all analyses, the statistical threshold was set at a voxel-wise threshold of *p* < 0.001, with a cluster-level family-wise error (FWE) correction at *p* < 0.05, corrected for multiple comparisons. We utilized the Automated anatomical labeling (AAL) atlas 3 [37] for labelling gray matter structures in VBM analyses.

##### Analysis of intrinsic resting-state functional connectivity

For rsFC data, seed-based connectivity (SBC) metrics were calculated in CONN. Two sets of analyses were conducted. First, a GLM was used to examine inter-regional connectivity alterations during the observation period across pre-defined regions. These included three key networks: the DMN, the SN, and the FPN. The analysis of these networks was conducted utilizing the standard anatomical seeds available in CONN. For the DMN, seed regions of interest were the medial prefrontal cortex (MPFC), the posterior cingulate cortex (PCC), and the bilateral lateral parietal lobules (L/R LP), for the SN the bilateral insula (L/R Insula), the anterior cingulate cortex (ACC), the rostral prefrontal cortex (L/R RPFC), and the supramarginal gyri (L/R SMG), as well as the bilateral lateral prefrontal cortex (L/R LPFC) for the FPN. Second, the bilateral amygdala connectivity maps were examined in relation to fatherly attachment based on PPAS total scores and subscales. Bivariate regressions were performed in CONN to assess significant inter-regional connectivity patterns with attachment measures at corresponding time points (e.g. PPAS T1 and amygdala connectivity T1). All results used a combination of a cluster-forming *p* < 0.001 voxel-level threshold, and a familywise corrected p-FDR < 0.05 cluster-size threshold, while correcting for multiple comparisons [38]. The AAL 3 atlas [37] was utilized to identify regions of interest and significant whole-brain findings across subjects.

## Results

### Whole-brain voxel-based analyses (VBM)

#### GMV decrease in the postpartum period

**Fig. 1:**
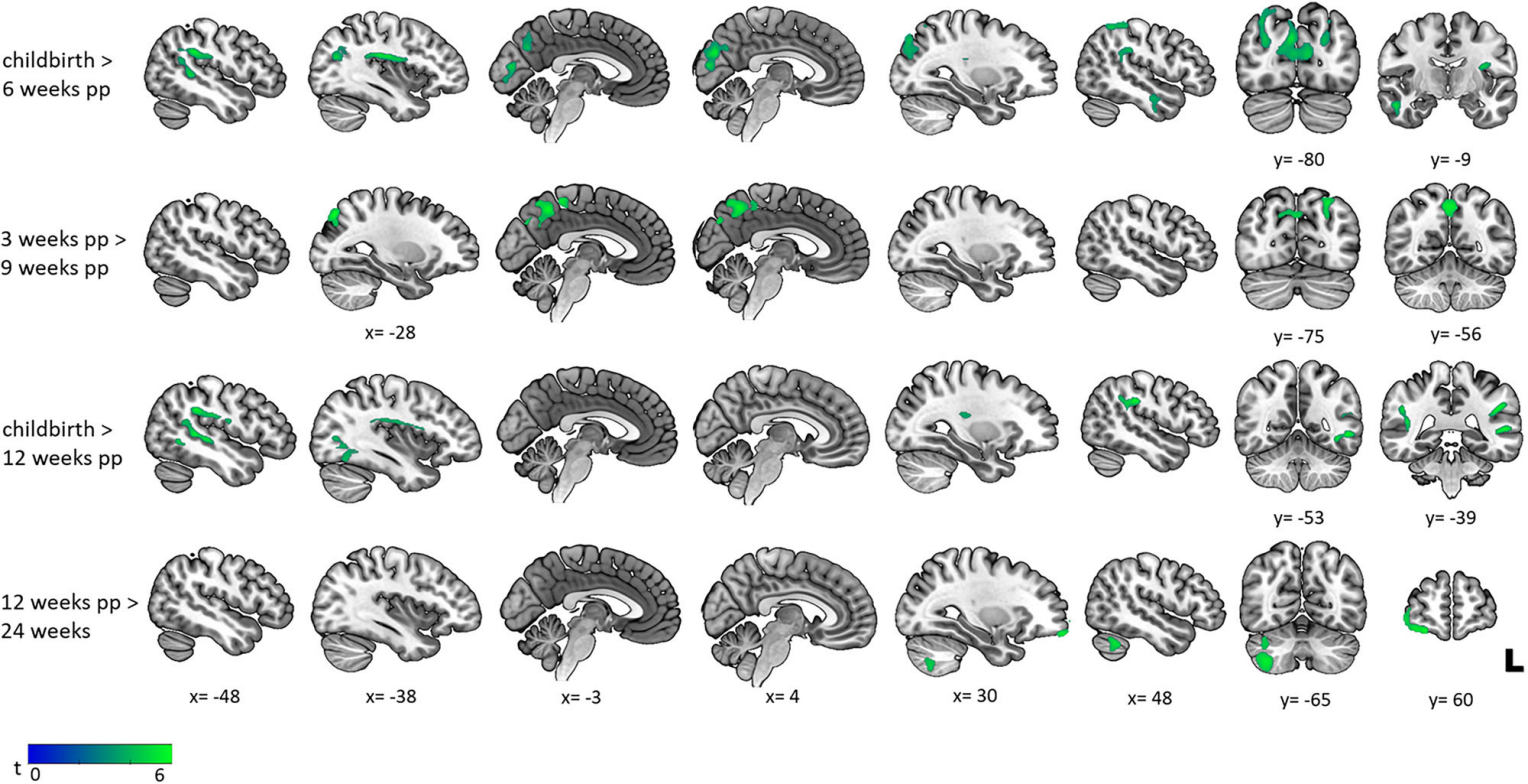
Longitudinal decreases in gray matter volume in fathers across the first 24 weeks postpartum. Significant gray matter volume reductions are shown for the following comparisons: childbirth vs. 6 weeks postpartum, 3 vs. 9 weeks postpartum, childbirth vs. 12 weeks postpartum, and 12 vs. 24 weeks postpartum. Reductions were most pronounced within the first 12 weeks postpartum across the bilateral frontal, temporal, parietal, and occipital cortices, with more circumscribed frontal and cerebellar decreases at 24 weeks postpartum (vs. 12 weeks).

Over the 24 weeks following childbirth, progressive reductions in GMV were observed, indicating a dynamic pattern of change (see Fig. 1). The most substantial alterations occurred in the first six weeks postpartum, with widespread reductions across multiple brain regions, including the bilateral parietal, temporal, frontal and occipital lobes, the temporo-parietal junction (TPJ), the insula, the SMG, as well as the left putamen. Please see Table 1 in SI for detailed information on regions, sizes and intensities of GMV changes. In the following weeks, GMV alterations were marked by less volume reductions but were likewise spread. Comparing weeks 9 and 12 postpartum to earlier time points, persistent reductions were seen in the bilateral occipital, frontal, temporal and parietal cortices, the insula, the TPJ, the SMG, and the left putamen. Additional reductions were found in the left cerebellum, the parahippocampal gyrus and the hippocampus (see Table 1 in SI). By 24 weeks after childbirth (vs. 12 weeks), GMV reductions had largely waned, with persistent decreases in the right cerebellum, the frontal gyrus, and the fusiform gyrus.

##### GMV increase in the postpartum period

**Fig. 2:**
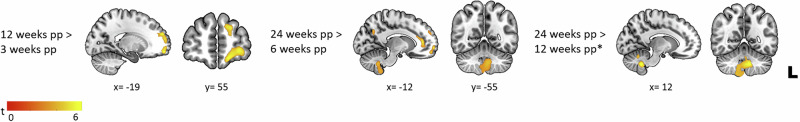
Longitudinal increases in gray matter volume in fathers across the first 24 weeks postpartum. Significant gray matter volume increases are shown for the following comparisons: 12 vs. 3 weeks postpartum, 24 vs. 6 weeks postpartum, and 24 vs. 3 weeks postpartum. Increases predominantly involved the left frontal gyri at 12 weeks postpartum, with additional occipital, anterior cingulate, cerebellar, and temporal involvement at 24 weeks postpartum. *performed using a cluster-forming threshold to *p* < 0.005, < 0.05 cluster-level FWE-correction.

Gradual increases in GMV were noted in local brain regions, highlighting a complex trajectory of primarily left-lateralized structural adaptation (see Fig. 2). At 12 weeks postpartum (vs. 6 weeks), GMV increases were identified in the left superior frontal gyrus, the medial and orbital regions of the frontal gyrus, and the rectal gyrus. These increases persisted when comparing the 24-week postpartum period to 6 and 12 weeks postpartum, with additional volume enlargements in the bilateral cerebellum, the left anterior cingulate cortex, the right cuneus, the calcarine fissure and surrounding cortex, as well as the occipital, and the temporal gyri (see Table 2 in SI).

### Resting-state connectivity changes of the triple-networks

Next, using an SBC analysis, we explored whether the transition to fatherhood entails alterations in the key functional brain networks: DMN, SN, FPN. Our observations revealed significant changes in inter-regional connectivity, involving both increases and decreases, across the postpartum period, particularly during the first 9 weeks.

#### The SN

**Fig. 3:**
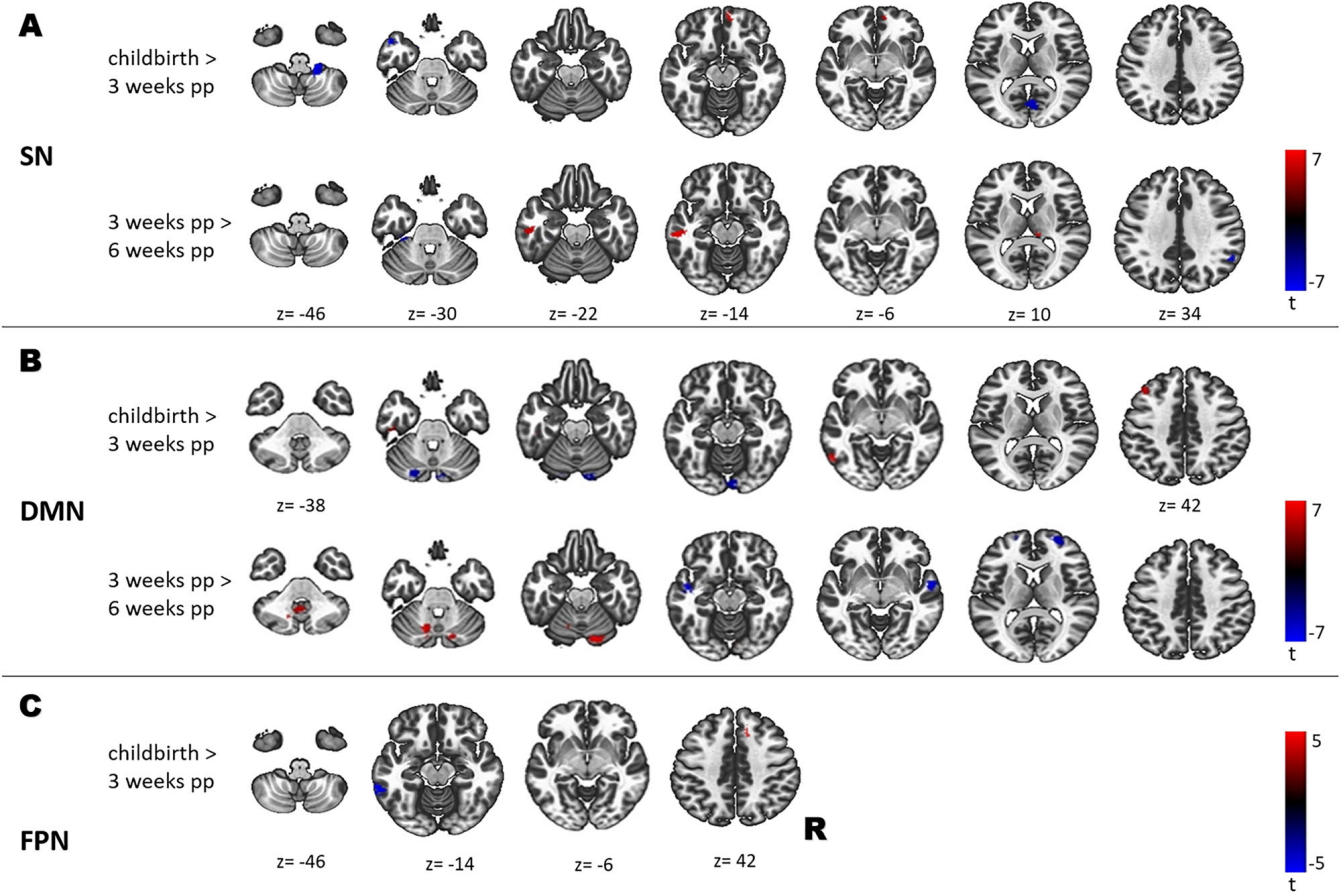
Longitudinal alterations in resting-state functional connectivity across large-scale networks in fathers during the early postpartum period. Significant seed-to-voxel connectivity increases and decreases are shown within three networks: **A** Salience Network (SN): connectivity changes involving frontal, parietal, posterior occipital, and cerebellar regions at childbirth vs. 3 weeks postpartum, with additional insular, thalamic, and cerebellar involvement at 3 vs. 6 weeks postpartum; **B** Default Mode Network (DMN): connectivity changes involving cerebellar, temporal, and occipital regions at childbirth vs. 3 weeks postpartum, with additional cingulate involvement at 3 vs. 6 weeks postpartum; **C** Fronto-Parietal Network (FPN): connectivity changes involving superior frontal, paracingulate, and temporal regions at childbirth vs. 3 weeks postpartum. SN salience network, DMN default mode network, FPN fronto-parietal network.

Our findings demonstrate that the most extensive postpartum functional reorganization occurs in the SN (see Fig. 3). Shortly after childbirth (vs. 3 weeks), we observed an increased SN connectivity with the frontal regions and decreased connectivity with the primary visual cortex (V1), the parietal cortices as well as the cerebellar regions (see Table 3 in SI). As the postpartum period progressed, cerebellar connectivity continued to decline, while connectivity between the insula and the thalamus increased (see Table 3 in SI). Functional reorganization of the SN peaked at 9 weeks postpartum, with sustained connectivity patterns extending to subcortical regions, including the caudate nucleus and putamen. These alterations stabilized by 12 weeks, accompanied by continued connectivity decreases with the cerebellar and frontal regions (see Table 3 in SI). From 12 to 24 weeks, functional changes became more widespread, marked by increased connectivity with the paracingulate gyrus, the cerebellar regions and the caudate, along with reduced connectivity with the superior parietal lobule (see Table 3 in SI).

##### The DMN

Within the DMN, we observed reduced connectivity with the cerebellar regions 3 weeks postpartum, accompanied by increased connectivity with the temporal and occipital areas, particularly in the left hemisphere (see Fig. 3 and Table 4 in SI). These connectivity changes reached their peak at 6 weeks, with previously observed alterations persisting and becoming more widespread. Notable additional changes included enhanced connectivity with the paracingulate and cingulate gyri. During the subsequent weeks, changes gradually diminished, with significant alterations limited to cerebellar (6 to 12 weeks) and thalamus connectivity (9 to 12 weeks). During the late postpartum period (12 to 24 weeks), increased connectivity with the paracingulate and cingulate gyri, the insula and central opercular cortices emerged, along with reduced connectivity with the superior parietal lobule (see Table 4 in SI).

##### The FPN

At 3 weeks postpartum, we observed reduced FPN connectivity with the temporal regions, alongside increased connectivity with the right superior frontal and paracingulate gyri (see Fig. 3 and Table 5 in SI). As the postpartum period progressed (3 to 6 weeks), these alterations diminished, with persistent reductions to the frontal gyri. Between 9 and 12 weeks, the connectivity changes reemerged, marked by increased connectivity with the posterior temporal regions and the cerebellum, indicating ongoing functional adaptation. No further FPN connectivity changes were observed during the late postpartum period (12 to 24 weeks).

### Neurocorrelates of paternal attachment

#### Associations with GMV

To complement our wider examination of GMV and large-scale neural network dynamics in the paternal brain, we examined potential neurocorrelates of paternal caregiving. First, we used multiple regression analyses to examine whether whole-brain GMV was related to concurrent PPAS total scores and subscales at each time point, and whether changes in GMV between time points (e.g., 3 to 6 weeks postpartum) predicted PPAS scores. No significant associations were found at any time point or for GMV changes, even at less conservative thresholds (voxel-level *p* < 0.001 FWE cluster-corrected, or *p* < 0.005 uncorrected).

##### Associations with inter-regional amygdala connectivity

**Fig. 4:**
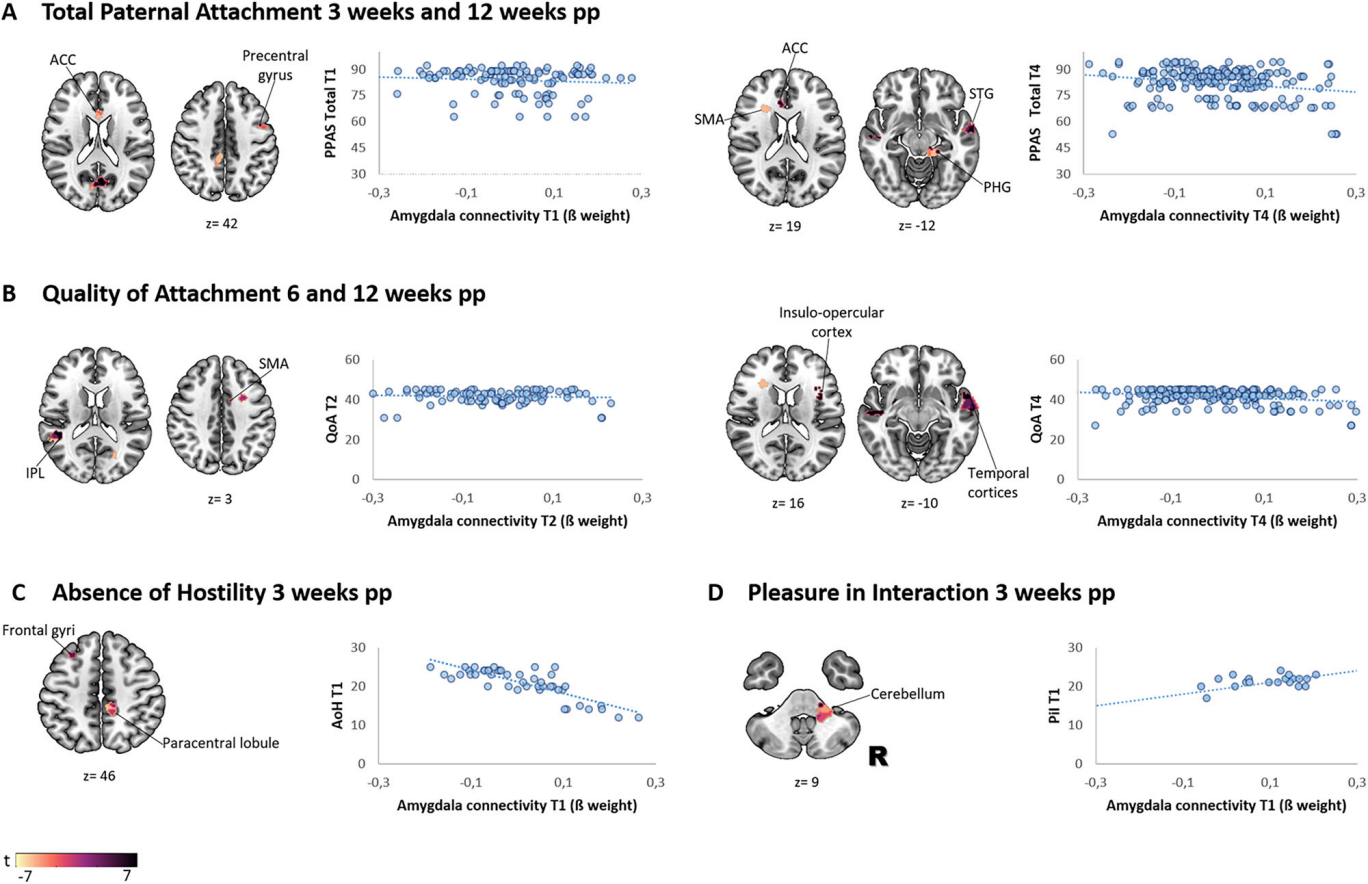
Associations between inter-regional amygdala connectivity and paternal attachment scores across the first 12 weeks postpartum. Shown are subject-specific beta values (Fisher Z-transformed correlation coefficients) representing the association between inter-regional amygdala connectivity (x-axis) and paternal attachment scores (y-axis). Each point reflects the extracted connectivity estimate from a significant amygdala cluster at the corresponding time point. Associations are displayed for: **A** PPAS total scores at 3 and 12 weeks postpartum; **B** Quality of Attachment scores at 6 and 12 weeks postpartum; **C** Absence of Hostility at 3 weeks postpartum; **D** Pleasure in Interaction at 3 weeks postpartum. pp postpartum, PPAS paternal postnatal attachment scale, QoA quality of attachment, AoH absence of hostility, PiI pleasure in interaction, ACC anterior cingulate cortex, SMA supplementary motor area, STG superior temporal gyrus, PHG parahippocampal gyrus, IPL inferior parietal lobule.

Finally, we performed a bivariate regression analysis to examine the relationship between inter-regional amygdala connectivity and paternal attachment scores. Significant associations were observed for total PPAS and its subscales within the first 12 weeks postpartum (see Fig. 4 and Supplementary Tables 6–9), with no significant findings at 24 weeks.

Associations with total attachment scores were observed as early as 3 weeks postpartum, including significant connectivity patterns involving the cingulate, parietal, occipital, and sensorimotor cortices (see Supplementary Table 6). In the subsequent weeks, new links to the hippocampal regions (6 weeks), the thalamus, the precuneus, and the postcentral gyrus (9 weeks) emerged (see Supplementary Table 6). By 12 weeks, these associations expanded to include the insular and cerebellar regions, the temporal gyrus, and the parahippocampal gyrus (see Supplementary Table 6).

Among the subscales, Quality of Attachment showed the strongest connectivity associations. At 3 weeks postpartum, increased thalamus connectivity was noted, which expanded to include the parietal and supracalcarine cortices, the supplementary motor cortex, and the inferior temporal gyrus by week 6 (see Supplementary Table 7). By weeks 9 and 12, these patterns extended to the hippocampal and cerebellar regions, as well as the temporal cortices and insula (see Supplementary Table 7). Absence of Hostility demonstrated consistent negative connectivity associations at 3, 9 and 12 weeks postpartum, involving the frontal regions, the paracentral lobule, the cingulate gyrus, and the operculum (see Supplementary Table 8). Finally, Pleasure in Interaction was found to be linked to cerebellar connectivity in the first 3 weeks, with expanding links to the central opercular cortex and superior temporal gyrus by 9 and 12 weeks (see Supplementary Table 9).

## Discussion

To investigate the adaptations of the paternal brain following childbirth, the present study sought to examine structural and functional connectivity changes in new fathers over a 24-week postpartum period. The findings reveal early postpartum morphological alterations characterized by decreasing GMV, followed by region-specific increases in the subsequent weeks. Significant functional reorganization within the DMN, SN, and FPN was observed during the first 12 weeks postpartum, with enhanced engagement of higher-order cognitive and emotional processing areas. Additionally, we identified associations between inter-regional amygdala connectivity and paternal attachment, including notable subscale-specific patterns in the hippocampal, the frontal and mentalizing regions. Collectively, the findings highlight a clear trajectory of paternal neuroplasticity, suggesting a brain adaptation during early fatherhood that might have had some evolutionary advantage.

### Paternal neuroplasticity during the first 24 weeks after childbirth

#### Morphological changes

Consistent with the notion of the “parental brain network” associated with human caregiving [30], we observed dynamic GMV alterations in the cortical (e.g. the bilateral TPJ, the OFC, the prefrontal cortices, the inferior parietal lobule, and insula) and subcortical areas (e.g. the putamen, parahippocampal gyrus, and hippocampus), encompassing networks involved in empathy, mentalizing, and executive control. These changes followed a pattern of GMV reductions during the early postpartum period, peaking within the first six weeks before gradually tapering. Our findings align with prior studies reporting paternal postpartum reductions in the insula, the fusiform gyri [18], and the cingulate cortex [18, 39], as well as the visual areas [20]. Additionally, the later postpartum period (12 and 24 weeks) was found to be characterized by increases in volume in the bilateral cerebellar and cingulate regions, as well as the frontal and occipital cortices.

Interestingly, the pattern of paternal GMV alterations contrasts with the maternal postpartum restructuring, which exhibits far greater and mostly increasing volume changes [3, 4]. While both still follow a similar temporal trajectory, with most significant alterations soon after childbirth, the underlying mechanisms are fundamentally different. In mothers, neuroplasticity is driven by substantial pregnancy-induced GMV loss, followed by regeneration during the postpartum period [3, 4]. During the first six weeks following childbirth, these changes are thought to be directly influenced by hormonal fluctuations, with the subsequent alterations being shaped by the demands of motherhood itself [40]. In contrast, fathers do not undergo these profound endocrinological shifts. Structural changes of the paternal brain may instead result from a confluence of factors: the adaption to fatherhood itself and experience-driven neuroplasticity. Indeed, the regions affected by these morphological changes likely play a vital role in the adaption process, while others aid the maintenance of conscious aspects of parenting. For instance, early changes within the empathy (i.e. insula-cingulate regions) and mentalizing networks (e.g.. the TPJ, the superior temporal gyrus) may enhance paternal attunement to their child’s state and intentions [31, 41], while noted changes in the “mirror” network (i.e. supplementary motor area, the IPL and frontal gyri) likely facilitate the embodied simulation of its actions [42]. Changes in the later postpartum period (i.e. 12 and 24 weeks), however, are characterized by localized increases in emotion-regulation network structures and stabilization of GMV reductions, likely reflecting the fine-tuning of higher-order functions like affect regulation, inhibition, and planning [32]. This trajectory highlights a progressive adaptation, with early structural reorganization supporting the adaption to fatherhood, whereas later adaptations help refine essential caregiving skills. Although further research is needed to substantiate this hypothesis, the observed temporal patterns support this assumption.

#### Functional connectivity alterations of the DMN, the SN, and the FPN

Beyond the morphological changes, our findings highlight functional reorganization within the networks (the DMN, SN, and FPN) critical for interconnected cognitive processes [43] and caregiving behaviors [30].

In line with the observed GMV changes in affect regulation and mentalizing regions, the SN was found to undergo the most substantial reorganization throughout the postpartum period. The network plays a key role in detecting relevant stimuli, processing emotions [44], and dynamically switching between the DMN and FPN to enable appropriate responses [32]. As soon as 3 weeks after childbirth, we found enhanced SN connectivity with the cerebellar and frontal regions, likely reflecting the initial caregiving demands. Over time, the connectivity was found to shift toward the hubs involved in affective processing and regulation, subsequently extending to the subcortical regions (e.g., insula) while connections with the frontal and cerebellar regions decreased. Concurrent reductions in connectivity within the sensory regions, such as the occipital cortices, suggest a shift from sensory to enhanced emotional processing during early caregiving [45, 46]. Interestingly, the DMN, essential for internal processes like mentalizing [47], exhibited similar reorganization patterns during early fatherhood. These alterations were marked by increased connectivity with regions associated with affective and cognitive processing (e.g., PCC-insula, MPFC-anterior cingulate) and decreased connectivity with multisensory regions (e.g., insula-angular gyrus) [26, 27]. The FPN, which supports higher-order cognitive functions [48], exhibited subtler yet persistent focal alterations, involving the temporal, frontal and paracingulate gyri. Consistent with the temporal trajectories observed in the DMN and SN, these changes stabilized after 12 weeks, underscoring the adaptive neuroplasticity processes during early fatherhood.

These results reveal a consistent pattern across the networks, characterized by a shift from sensory processing to progressively stronger connectivity with higher-order cognitive and socio-emotional networks. Whereas early reorganization processes likely reflect the initial demands of fatherhood, later engagement of the “parental caregiving network” [30], involving affect regulation, cognitive control [45, 46, 49], and mentalizing regions [50], may mirror the acquisition of caregiving behaviors. Our observations align with prior findings on greater connectivity in paternal socio-cognitive and affective processing regions [22, 23, 26], reinforcing the idea that heightened connectivity in parenting-related regions emerges as a result of new fatherhood [26, 27]. In line with this, converging functional neuroimaging evidence suggests that caregiving experience itself, rather than biological relatedness per se, drives neural adaptations within parental brain networks. Studies of primary-caregiving homosexual fathers and other committed non-biological caregivers demonstrate the engagement of the “parental caregiving network” encompassing affective, mentalizing, and amygdala-centered connectivity comparable to what is observed in biological parents [26, 30]. Our findings are consistent with this notion, suggesting that the observed network reorganization reflects experience-dependent caregiving adaptations. Notably, the functional reorganization processes also coincide with observed GMV alterations, beginning with initial adaptations to the new demands, followed by localized refinement. This pattern is further evident in our connectivity data, where by 12 to 24 weeks postpartum, both the DMN and SN showed solely sustained enhanced connectivity with regions involved in affect regulation, reward processing, and cognitive flexibility (e.g., insula, precuneus, caudate). Functional alteration processes during the late postpartum period are thus marked by ongoing neural reorganization and, in line with maternal findings, are underpinned by the engagement of reward-related motivation [51].

##### Amygdala connectivity and associations to fatherly attachment

The amygdala has consistently been highlighted as a key component of the human parental brain, driving parental vigilance and reward from attachment bond [30]. It has also been directly linked to paternal involvement [26, 27] as well as maternal attachment [4]. In line with these observations, we found significant associations between inter-regional amygdala connectivity and both the total PPAS score and its subscales during the first 12 weeks postpartum. These associations were evident as early as three weeks postpartum and became increasingly distinct and persistent over time. In the early postpartum period, enhanced amygdala-cingulate connectivity (PPAS total) and amygdala-cerebellar connectivity (Pleasure in Interaction) were observed, suggesting the involvement of neural circuits that prioritize processing emotional and caregiving-related stimuli during early bonding [52]. Moreover, the associations of Absence of Hostility and reduced connectivity with the cingulate and frontal gyri likely reflect the role of emotion regulation and inhibitory control [53] in mitigating hostile responses during child-rearing. In the subsequent weeks, persisting subscale-specific patterns emerged, marked by increased amygdala-hippocampal connectivity for both Total and Quality of Attachment. The enhanced connectivity between these reciprocally connected regions has been associated with improved memory for emotional events [54] and may, within the context of new fatherhood, reflect the consolidation of attachment-related experiences, thereby facilitating the internalization of these interactions. While the exact mechanisms by which the amygdala contributes to parental attachment remain to be fully elucidated, the findings underscore its role in the neurobiological foundations of fatherhood with its co-wiring to parenting-related structures likely fostering early bonding [30].

### Limitations

A major limitation of this study is its observational design, which does not explore the specific neuronal mechanisms underlying the observed changes in brain volume and functional connectivity in the context of new fatherhood. Further, the lack of a control group, or a pre-childbirth measurement, prevents direct comparisons of postpartum-specific changes in the male brain. Additionally, three participants were not first-time fathers, limiting the ability to draw definitive conclusions or to examine potential differences in neuroplasticity related to previous parenting experience. These limitations, however, are mitigated by the study’s design, which strengthens the reliability and validity of the observed findings. The longitudinal approach, with stringent time intervals, offers a robust framework for tracking neuroplasticity changes over time. This is further substantiated by repeated measurements at these intervals, which reduce the potential for random fluctuations. Moreover, the affected regions do not appear to be incidental, rather, they involve areas previously implicated in, or thought to be critical for, parenting and caregiving behaviors. This is substantiated by the association between amygdala connectivity and paternal attachment. Nonetheless, future neuroimaging studies should aim to address these limitations while providing insights into the neural reorganization in new fathers throughout pregnancy and into the postpartum period. A comparison between fathers and non-fathers with respect to their neural, neuroendocrine, and epigenetic mechanisms can further deepen our knowledge of adaptions during the transition to fatherhood. Indeed, compared to the well-established knowledge of maternal changes, our understanding of the neurobehavioral basis of paternity remains limited with only a handful of studies affording insights into male endocrinological [8, 9] and neural adaptions [7, 18, 20] during pregnancy and after childbirth. Future research should aim to bridge this gap by adopting a more integrative approach that considers both parents equally, moving beyond the traditional focus on mothers.

## Conclusion

In conclusion, our findings reveal significant morphological and functional connectivity changes in the male brain following childbirth, with the first 6–9 weeks postpartum emerging as a critical period for paternal neuroplasticity. These alterations follow a temporal pattern characterized by rapid, progressive adaptations to the new demands of fatherhood, followed by later refinements to support caregiving and attachment-related functions. Although the exact mechanisms behind these neural changes are unclear, and beyond the scope of this study, the affected regions and their temporal trajectory suggest a direct influence of childbirth, likely reflecting an evolutionary adaptation within the context of new fatherhood. This is particularly highlighted by the restructuring of regions critical for parenting [30] and the amygdala’s significant association with fatherly attachment, as the early postpartum period is a crucial window not only for paternal neural reorganization, but also for the development of paternal attachment itself.

## Supplementary information

Supplementary Material

## Data Availability

The data presented in this study are not publicly available due to privacy restrictions, but are available on request.
