# Supplementary material for: The paternal brain: longitudinal insights into structural and functional plasticity and attachment over 24 weeks postpartum
Source: Transl Psychiatry. 2026 May 14;16:247. doi: 10.1038/s41398-026-04082-7 (PMC13176344; doi:10.1038/s41398-026-04082-7)
Supplement: Supplementary file 1 — Supplementary Material [file 41398_2026_4082_MOESM1_ESM.docx]

**Supporting Information**

**Recruitment procedure**

Beginning within one to six days of childbirth, we recruited 26 new fathers at University Hospital Aachen. The exclusion criteria included current or past neurological or psychiatric disorders, use of antidepressant or antipsychotic medication, substance dependency, severe obesity (BMI > 35), serious neonatal conditions (e.g., brain damage, chromosomal abnormalities, major congenital malformations), preterm birth (<29 weeks gestational age), and standard MRI contraindications. After obtaining informed consent, participants were first screened for prenatal depression and were included only if clinical depression was not diagnosed. Additionally, clinical and anamnestic evaluations were conducted, gathering demographic details, pregnancy- and birth-related information, as well as individual and family psychiatric histories.

**Power analysis and sample size considerations**

We conducted an a priori power analysis to determine the sample size required to detect longitudinal changes in brain structure and connectivity. Assuming a medium effect size (Cohen’s f = 0.25), commonly reported for longitudinal neuroplasticity effects, G*Power analyses (Faul et al., 2007) indicated that 20-24 participants would be sufficient to detect significant within-subject effects over six repeated measurements (α = .05, power = .80). Our final sample of 25 fathers thus provided adequate power for the planned analyses.

**MRI acquisition and preprocessing**

MRI acquisitions were performed on a 3 Tesla Prisma MR Scanner (Siemens Medical Systems, Erlangen, Germany) located in the Medical Faculty of University Hospital Aachen. Structural T1- weighted images were acquired using 3-dimensional magnetization-prepared rapid acquisition gradient echo imaging sequence (4.12 min; 176 slices, TR = 2300 ms, TE = 1.99 ms, TI= 900 ms, FoV = 256 x 256 mm, flip angle= 9°, voxel resolution = 1x1x1mm^3^). Whole-brain echoplanar images (EPIs) were acquired during rest and eyes closed with the following acquisition parameters: 6.6 min; 36 slices, TR= 2200 ms, TE= 30 ms, FoV = 200 x 200 mm, flip-angle= 90°, number of volumes= 300, voxel-size= 3.1 x 3.1 x 3.1 mm^3^. All images were examined for structural abnormalities, as well as scanner and motion artifacts. If artifacts were detected, the imaging process was repeated.

Anatomical MRI data were preprocessed using the Computational Anatomy Toolbox (CAT12.8.2 Version r2170) and statistical parametric mapping (SPM) 12 toolbox, implemented in Matlab 2019b (MathWorks). Images of each subject were preprocessed according to the longitudinal protocol in CAT12 (Gaser et al., 2023). For resting-state data, preprocessing was conducted utilizing the default preprocessing pipeline of CONN functional connectivity toolbox (CONN, version 22.a, https://www.nitrc.org/projects/conn) implemented in SPM12 and Matlab 2019b (MathWorks).

**Voxel Based Morphometry (VBM)**

All images were affine-registered to standard tissue probability maps by adjusting individual head positions and orientations, then transformed into Montreal Neurological Institute (MNI) space. The structural T1-weighted images from each time point were spatially normalized to MNI space (resampled to a voxel size of 1.5×1.5×1.5 mm) and segmented into gray matter (GM), white matter (WM), and cerebrospinal fluid (CSF). The images were visually checked for any segmentation or registration errors. In accordance with the CAT12 toolbox manual (Gaser et al., 2023), a homogeneity check of the unsmoothed data showed no outliers, and the GMVs of all participants were included in the subsequent analyses. Lastly, the modulated GMV was smoothed using an 8-mm full-width at half-maximum (FWHM) Gaussian kernel.

**Resting-state functional MRI data processing**

Preprocessing was conducted utilizing the default preprocessing pipeline of CONN, including realignment with correction of susceptibility distortion interactions, slice time correction, direct segmentation, normalization into MNI-template space, and smoothed utilizing a spatial convolution with a Gaussian Kernel of 8 mm full width half maximum (FWHM). Potential outliers were identified using ART as acquisitions with frame wise displacement above 0.5 mm or global BOLD signal changes above 3 standard deviations. The functional data were then denoised using the standard denoising pipeline, including scrubbing, based upon potential outliers detected, and the regression of potential confounding effects of motion parameters. Structural images were co-registered to functional data and segmented into gray matter, white matter and CSF. Utilizing the anatomical component-based noise correction procedure (aCompCor), potential confounding effects were regressed out from the blood oxygen level-dependent (BOLD) signal. Parameters from the aforementioned steps, i.e. realignment, scrubbing, white matter and CSF signal parameters, were used as first-level covariates. Seed-based connectivity maps (SBC) were estimated characterizing the patterns of FC with 164-HPC-ICA networks (Nietro-Castano & Whitfield- Gabrieli, 2019). FC strength was represented by Fisher-transformed bivariate correlation coefficients from a weighted general linear model (weighted-GLM), defined separately for each pair of seed, modelling the association between their BOLD signal timeseries. These values of each voxel across the entire brain indicate the relative level of functional connectivity with each seed (Whitfield-Gabrieli et al., 2010) and are subsequently utilized for the second-level analysis.

**Analyses of Seed-based connectivity (SBC) maps**

For the second-level analysis, we performed a GLM. For each individual voxel a separate GLM was estimated across pre-defined regions, with first-level connectivity measures at this voxel as dependent variables subject-level identifiers as independent variables. To account for potential confounding effects of paternal age on functional connectivity, paternal age was included as a covariate of no interest in the GLM. Seed regions of interest included three key networks: the DMN, the SN, and the FPN. For the DMN, seed regions of interest were the medial prefrontal cortex (MPFC), the posterior cingulate cortex (PCC), and the bilateral lateral parietal lobules (L/R LP), for the SN the bilateral insula (L/R Insula), the anterior cingulate cortex (ACC), the rostral prefrontal cortex (L/R RPFC), and the supramarginal gyri (L/R SMG), as well as the bilateral lateral prefrontal cortex (L/R LPFC) for the FPN.

Voxel-level hypotheses were evaluated using multivariate parametric statistics with random-effects across subjects and sample covariance estimation across multiple measurements. Inferences were performed at the level of individual clusters (groups of contiguous voxels) and significant effects were pursued with t-contrasts. Cluster-level inferences were based on parametric statistics from Gaussian Random Field theory (Nieto-Castanon, et al., 2020; Worsley et al., 1996). All results were thresholded using a combination of a cluster-forming p < 0.001 voxel-level threshold, and a familywise corrected p-FDR < 0.05 cluster-size threshold (Chumbley et al., 2010). For each individual voxel a separate GLM was estimated, with first-level connectivity measures at this voxel as dependent variables.

**Multivariate Regression**

To examine whether changes in whole-brain GMV were associated with paternal attachment, we performed multivariate pattern analyses using PRoNTo 3.0 [Desikan et al., 2006), a toolbox designed for neuroimaging pattern recognition. Whole-brain voxel-wise GMV images were used to predict PPAS total scores and subscales (Quality of Attachment, Absence of Hostility, and Pleasure in Interaction). For analyses of GMV changes across the postpartum period, GMV change images were created by subtracting earlier from later time points for each subject (e.g., 6 weeks minus 3 weeks) using the ImCalc function in SPM12. Kernel ridge regression was applied to these images to predict paternal attachment scores, controlling for age. A leave-one-out cross-validation approach was used, following the PRoNTo user manual recommendations. Model performance was evaluated using Pearson’s correlation (r) between observed and predicted scores, explained variance (R²), and root mean squared error (RMSE). Permutation testing (1000 repetitions) was performed to assess statistical significance. For completeness, age was regressed out prior to modeling, and all GMV images were mean-centered and normalized.

**Table S1.** Brain regions showing gray matter volume decreases in postpartum men throughout the postpartum period (t-contrast from random-effects GLM, p < .001, cluster-level FWE correction, unless otherwise specified).

| **Anatomical Region** | Brodmann | Side | Size | T | x | y | z |  |
| --- | --- | --- | --- | --- | --- | --- | --- | --- |
| **childbirth > 6 weeks** |  |  |  |  |  |  |  |  |
| Supramarginal gyrus, insula, angular gyrus, rolandic operculum, middle occipital gyrus, superior temporal gyrus, postcentral gyrus, inferior parietal gyrus, inferior frontal gyrus pars opercularis, middle temporal gyrus, putamen, precentral gyrus, Heschl’s gyrus | 13, 40, 39 | L | 2438 | 5.65 | -33 | -25.5 | 24 |  |
| Supramarginal gyrus, insula, rolandic operculum, superior temporal gyrus | 13,40, 2, 41 | R | 610 | 5.38 | 40.5 | -31.5 | 25.5 |  |
| Cuneus, calcarine fissure and surrounding cortex, precuneus, superior occipital gyrus, middle occipital gyrus, superior parietal gyrus, inferior parietal gyrus, lingual gyrus | 18, 7, 19, 17, 31, 23 | L/R | 2887 | 5.33 | 4.5 | -78 | 27 |  |
| Inferior temporal gyrus, middle temporal gyrus | 20, 21 | R | 419 | 4.84 | 52.5 | -10.5 | -24 |  |
| Middle temporal gyrus, superior temporal gyrus, angular gyrus, supramarginal gyrus | 40, 22, 13 | L | 583 | 4.81 | -48 | -42 | 4.5 |  |
| Superior parietal gyrus, inferior parietal gyrus, angular gyrus, postcentral gyrus | 40, 7, 2 | R | 570 | 4.52 | 49.5 | -49.5 | 57 |  |
| Superior occipital gyrus, middle occipital gyrus, superior parietal gyrus, cuneus | 19, 7, 18 | R | 719 | 4.36 | 30 | -81 | 40.5 |  |
| **3 weeks > 6 weeks** |  |  |  |  |  |  |  |  |
| Superior parietal gyrus, postcentral gyrus, inferior parietal gyrus, angular gyrus, supramarginal gyrus | 40, 7, 2, 1, 4, 6, 3, 5 | R | 1155 | 5.05 | 48 | -48 | 57 |  |
| Middle occipital gyrus, superior occipital gyrus | 19, 7 | R | 478 | 4.69 | 28.5 | -76.5 | 31.5 |  |
| Middle occipital gyrus, superior parietal gyrus, superior occipital gyrus, inferior parietal gyrus | 7, 19 | L | 723 | 4.39 | -24 | -72 | 40.5 |  |
| Supramarginal gyrus, insula, rolandic operculum, superior temporal gyrus | 13, 40 | L | 651 | 6.51 | -31.5 | -28.5 | 22.5 |  |
| Precuneus, cuneus | 7 | L/R | 567 | 3.91 | -3 | -63 | 55.5 |  |
| **3 weeks > 9 weeks** |  |  |  |  |  |  |  |  |
| Superior parietal gyrus, middle occipital gyrus, inferior parietal gyrus, superior occipital gyrus | 7, 19 | L | 435 | 4.72 | -28.5 | -78 | 46.5 |  |
| Precuneus, cuneus, paracentral lobule, supplementary motor area, superior occipital gyrus | 7, 5, 6, 4, 31 | L/R | 1766 | 4.56 | -3 | -54 | 51 |  |
| **childbirth > 12 weeks** |  |  |  |  |  |  |  |  |
| Supramarginal gyrus, insula, superior temporal gyrus, rolandic operculum, inferior parietal gyrus, middle temporal gyrus, angular gyrus | 13, 2, 40, 41 | R | 790 | 5.26 | 46.5 | -30 | 31.5 |  |
| Middle temporal gyrus, superior temporal gyrus | 22, 21, 40, 13 | L | 692 | 5.11 | -55.5 | -40.5 | 9 |  |
| Middle temporal gyrus, fusiform gyrus, inferior temporal gyrus, middle occipital gyrus, inferior occipital gyrus | 37 | L | 617 | 5.07 | -52.5 | -54 | -3 |  |
| Supramarginal gyrus, postcentral gyrus, insula, rolandic operculum, putamen, inferior frontal gyrus opercular part, inferior parietal gyrus, superior temporal gyrus, precentral gyrus | 13, 40, 43, 6, 4 | L | 1437 | 5.00 | -48 | -39 | 31.5 |  |
| Parahippocampal gyrus, lingual gyrus, cerebellum 4_5, hippocampus | 28, 35 | L | 130 | 4.58 | -18 | -30 | -13.5 |  |
| Supramarginal gyrus, insula, rolandic operculum | 13,40 | R | 156 | 4.42 | 34.5 | -25.5 | 19.5 |  |
| **12 weeks > 24 weeks** |  |  |  |  |  |  |  |  |
| Middle frontal gyrus, superior frontal gyrus | 11, 10 | R | 609 | 4.50 | 27 | 63 | -18 |  |
| Cerebellum 8, cerebellum crus 1, cerebellum crus 2, cerebellum 7b, cerebellum 6, fusiform gyrus |  | R | 1865 | 4.25 | 34.5 | -64.5 | -51 |  |
|  |  |  |  |  |  |  |  |  |

**Table S2.** Brain regions showing gray matter volume increase in postpartum men throughout the postpartum period (t-contrast from random-effects GLM, p < .001, cluster-level FWE correction, unless otherwise specified).

| **Anatomical Region** | **Brodmann** | **Side** | **Size** | **T** | **x** | **y** | **z** |
| --- | --- | --- | --- | --- | --- | --- | --- |
| **12 weeks > childbirth** |  |  |  |  |  |  |  |
| Superior frontal gyrus, superior frontal gyrus orbital part, middle frontal gyrus, middle frontal gyrus medial orbital part, middle frontal gyrus orbital part, superior frontal gyrus medial part, gyrus rectus | 10, 11 | L | 1740 | 4.84 | -18 | 62 | -6 |
| **12 weeks > 3 weeks** |  |  |  |  |  |  |  |
| Superior frontal gyrus, middle frontal gyrus, superior frontal gyrus medial orbital part, superior medial frontal gyrus, inferior frontal gyrus pars orbitalis | 10, 11 | L | 889 | 3.80 | -16 | 57 | -15 |
| Superior frontal gyrus, middle frontal gyrus, superior frontal gyrus medial part | 10 | L | 797 | 4.50 | - 19.5 | 49.5 | 24 |
| **12 weeks > 6 weeks** |  |  |  |  |  |  |  |
| Superior frontal gyrus, middle frontal gyrus, superior frontal gyrus medial part | 10, 9 | L | 754 | 4.81 | -18 | 57 | 24 |
| **24 weeks > childbirth** |  |  |  |  |  |  |  |
| Superior frontal gyrus, superior frontal gyrus orbital part, middle frontal gyrus, middle frontal gyrus orbital part, superior frontal gyrus medial orbital part, superior frontal gyrus medial part, gyrus rectus, inferior frontal gyrus pars orbitalis | 10, 11 | L | 1714 | 5.77 | -13.5 | 64.5 | -4.5 |
| Superior frontal gyrus orbital part, superior frontal gyrus medial part, superior frontal gyrus, middle frontal gyrus orbital part, superior frontal gyrus medial orbital part, gyrus rectus | 10, 11 | R | 488 | 4.69 | 10.5 | 60 | -18 |
| **24 weeks > 3 weeks** |  |  |  |  |  |  |  |
| Superior frontal gyrus, middle frontal gyrus, superior frontal gyrus orbital part, middle frontal gyrus orbital part, superior frontal gyrus medial orbital part, superior frontal gyrus medial part, gyrus rectus, inferior frontal gyrus pars orbitalis | 10, 11 | L | 1880 | 5.80 | -12 | 58.5 | -15 |
| Superior frontal gyrus orbital part, superior frontal gyrus medial part, superior frontal gyrus, miffle frontal gyrus orbital part, superior frontal gyrus medial orbital part, gyrus rectus | 10, 11 | R | 464 | 4.95 | 16.5 | 58.5 | -10.5 |
| **24 weeks > 6 weeks** |  |  |  |  |  |  |  |
| Middle frontal gyrus, superior frontal gyrus, superior frontal gyrus orbital part, middle frontal gyrus orbital part, superior frontal gyrus medial part, superior frontal gyrus medial orbital part, inferior frontal gyrus triangular part, gyrus rectus | 10, 11, 9, 46 | L | 1708 | 5.98 | -15 | 63 | -3 |
| Middle occipital gyrus, superior occipital gyrus, cuneus, calcarine fissure and surrounding cortex, inferior occipital gyrus, lingual gyrus, fusiform gyrus, middle temporal gyrus | 18, 17, 19, 31, 7, 39 | R | 1068 | 5.35 | 24 | -79.5 | 19.5 |
| Anterior cingulate, paracingulate gyri | 32, 24, 10 | L | 588 | 5.10 | -10.5 | 39 | 9 |
| Cerebellum_9, vermis_9 |  | L/R | 1344 | 4.42 | -6 | -54 | -43.5 |
| **24 weeks > 12 weeks (p < .005)** |  |  |  |  |  |  |  |
| Cerebellum 9, vermis 9, cerebellum 8, cerebellum 8, cerebellum 4_5, vermis 6, vermis 8, vermis 4_5, vermis 10 | Dentate | L /R | 2045 | 4.18 | -12 | -54 | -42 |

**Table S3.** Functional connectivity maps of the SN in postpartum men throughout the postpartum period (t-contrasts from random-effects GLM, p < .05, cluster-size p-FDR corrected; voxel threshold: p < .001 p-uncorrected).

| **Network** | **Anatomical Region** | **Connectivity** | | **Side** | **Size** | **p- value** | **T** | **x** | **y** | **z** |
| --- | --- | --- | --- | --- | --- | --- | --- | --- | --- | --- |
|  | **Childbirth > 3 weeks** |  | |  |  |  |  |  |  |  |
| RPFC/ L | Cerebellum 8, cerebellum 7b | | increase | L | 72 | 0.013247 | 6.47 | -28 | -58 | -48 |
| RPFC/ R | Frontal pole, frontal medial cortex, paracingulate gyrus | | increase | R | 120 | 0.001196 | 5.11 | 10 | 52 | -10 |
|  | Temporal pole | | decrease | L | 72 | 0.012092 | -4.69 | -42 | 12 | -32 |
|  | Temporal pole, parahippocampal gyrus anterior division | | decrease | L | 64 | 0.014108 | -5.02 | -14 | 6 | -36 |
| SMG/ L | Intracalcerine cortex, lingual gyrus, supracalcerine cortex, vermis 4 5, cuneal cortex, vermis 6, precuneus, cerebellum 4 5, | | decrease | L/R | 505 | 0.000000 | -5.04 | 2 | -68 | 2 |
|  | Cerebellum 9, cerebellum 8, cerebellum 10 | | decrease | R | 292 | 0.000000 | -6.20 | 18 | -42 | -46 |
|  | **3 weeks > 6 weeks** | |  |  |  |  |  |  |  |  |
| Insula/ L | Frontal pole | | increase | R | 68 | 0.00944 | 4.89 | 18 | 42 | -10 |
|  | Middle frontal gyrus, inferior frontal gyrus pars triangularis | | decrease | L | 54 | 0.049645 | -4.62 | -36 | 26 | 18 |
| SMG/ L | Middle temporal gyrus posterior division, inferior temporal gyrus posterior division, superior temporal gyrus posterior division | | increase | L | 217 | 0.00035 | 5.65 | -46 | -20 | -14 |
|  | Middle temporal gyrus temporooccipital part, supramarginal gyrus posterior division, angular gyrus, superior temporal gyrus posterior division | | decrease | L | 124 | 0.001250 | -5.19 | -60 | -50 | 16 |
|  | Temporal fusiform cortex posterior division, cerebellum 4 5, parahippocampal gyrus | | decrease | L | 63 | 0.025874 | -4.97 | -30 | -32 | -28 |
|  | Angular gyrus, supramarginal gyrus posterior division | | decrease | R | 55 | 0.032680 | -6.41 | 54 | -50 | 32 |
| SMG/ R | Lingual gyrus, cerebellum crus 2, cerebellum crus 1, vermis 6, vermis 7 | | decrease | L/R | 67 | 0.029332 | -5.94 | 00 | -84 | -20 |
|  | Precentral gyrus, superior frontal gyrus, supplementary motor cortex | | decrease | R | 64 | 0.029332 | -4.71 | 14 | -12 | 72 |
|  | Thalamus | | increase | R | 50 | 0.040217 | 5.38 | 12 | -30 | 6 |
|  | Precentral gyrus, superior frontal gyrus | | decrease | L | 48 | 0.040217 | -5.83 | -16 | -12 | 70 |
|  | **6 weeks > 9 weeks** | |  |  |  |  |  |  |  |  |
| ACC | Insular cortex, frontal operculum cortex, inferior frontal gyrus pars opercularis, middle frontal gyrus | | decrease | L | 96 | 0.002458 | -5.56 | -28 | 20 | 10 |
|  | Cerebellum crus 2, cerebellum crus 1, cerebellum 7b, cerebellum 9 | | increase | R | 45 | 0.038976 | 4.97 | 38 | -68 | -44 |
| Insula/ L | Frontal pole, frontal medial cortex, paracentral gyrus | | decrease | R | 375 | 0.000000 | -6.44 | 16 | 50 | -18 |
|  | Frontal Pole, cingulate gyrus anterior division, subcallosal cortex | | decrease | L | 303 | 0.000000 | -5.71 | -14 | 34 | 00 |
|  | Frontal pole | | decrease | R | 294 | 0.000000 | -6.82 | 30 | 40 | 40 |
|  | Frontal pole | | decrease | L | 191 | 0.000009 | -5.31 | -20 | 42 | 50 |
| RPFC/ L | Caudate nucleus, subcallosal cortex, accumbens, putamen, | | increase | L | 347 | 0.000000 | 6.41 | -16 | 26 | -6 |
| SMG/ L | Thalamus, parahippocampal gyrus | | decrease | L | 101 | 0.006056 | -4.93 | -14 | -22 | -10 |
|  | Frontal pole | | increase | R | 63 | 0.028345 | 4.77 | 48 | 42 | 2 |
| SMG/ R | Frontal pole | | increase | R | 235 | 0.000023 | 5.23 | 48 | 44 | 00 |
|  | Frontal operculum cortex, insular cortex, inferior frontal gyrus pars triangularis | | increase | R | 104 | 0.004212 | 5.03 | 36 | 24 | 6 |
|  | Postcentral gyrus, supramarginal gyrus anterior division, precentral gyrus | | increase | R | 64 | 0.026945 | 4.82 | 64 | -14 | 32 |
|  | **9 weeks > 12 weeks** | |  |  |  |  |  |  |  |  |
| ACC | Supramarginal gyrus posterior division, angular gyrus, supramarginal gyrus anterior division, superior parietal lobule | | increase | L | 79 | 0.0125727 | 6.36 | -52 | -52 | 42 |
| Insula/ R | Cerebellum 4 5, vermis 4 5, lingual gyrus, vermis 3, cingulate gyrus posterior division | | decrease | L | 96 | 0.002814 | -4.76 | -2 | -44 | -2 |
| RPFC/ R | Frontal pole | | decrease | L/R | 139 | 0.000473 | -5.45 | 2 | 66 | 20 |
| SMG/L | Frontal pole | | decrease | L | 118 | 0.001463 | -4.93 | -36 | 60 | -14 |
|  | **12 weeks > 24 weeks** | |  |  |  |  |  |  |  |  |
| ACC | Paracingulate gyrus | | increase | L/R | 158 | 0.000131 | 4.91 | -4 | 46 | 14 |
|  | Frontal pole | | increase | R | 53 | 0.037342 | 4.54 | 8 | 60 | -18 |
| Insula/ L | Insular cortex, putamen | | increase | L | 126 | 0.001495 | 5.72 | -28 | 6 | 22 |
| Insula/ R | Precuneus, intracalcerine cortex, supracalcerine cortex | | increase | L/R | 352 | 0.000000 | 6.47 | 00 | -64 | 22 |
|  | Caudate | | increase | L | 94 | 0.000208 | 5.68 | -12 | 22 | 10 |
|  | Paracingulate gyrus | | increase | R | 89 | 0.002064 | 5.28 | 18 | 34 | 4 |
| RPFC/ R | Paracingulate gyrus, superior frontal gyrus, cingulate gyrus anterior division | | increase | L/R | 127 | 0.000784 | 4.59 | -12 | 12 | 48 |
|  | Middle temporal gyrus posterior division | | increase | L | 66 | 0.015116 | 4.96 | -66 | -34 | -6 |
| SMG/ L | Superior parietal lobule | | decrease | L | 67 | 0.034265 | -4.97 | -24 | -52 | 48 |
|  | Cerebellum 6 | | increase | R | 59 | 0.034265 | 4.92 | 22 | -62 | -20 |

Note. SN (Salience Network); L (left); R (right); ACC (anterior cingulate cortex); RPFC/ L (left rostral prefrontal cortex); RPFC/ R (right rostral prefrontal cortex); SMG/ L (left supramarginal gyrus); SMG/ R (right supramarginal gyrus)

**Table S4.** Functional connectivity maps of the DMN in postpartum men throughout the postpartum period (t-contrasts from random-effects GLM, p < .05, cluster-size p-FDR corrected; voxel threshold: p < .001 p-uncorrected).

| **Network** | **Anatomical Region** | **Connectivity** | | **Side** | **Size** | **p- value** | **T** | **x** | **y** | **z** |
| --- | --- | --- | --- | --- | --- | --- | --- | --- | --- | --- |
|  | **Childbirth > 3 weeks** |  | |  |  |  |  |  |  |  |
| LP_r | Vermis 8, cerebellum 8, Vermis 6, Vermis 9, cerebellum crus1, cerebellum 6, vermis 7, cerebellum 4 5, vermis 4 5, cerebellum cruz 2, cerebellum 9 | decrease | | L/R | 256 | 0.000014 | -5.16 | -6 | -58 | -28 |
|  | Middle temporal gyrus temporooccipital part, lateral occipital cortex inferior division, inferior temporal gyrus temporooccipital part | increase | | L | 93 | 0.008097 | 6.18 | -56 | -62 | -6 |
|  |  |  | |  |  |  |  |  |  |  |
| PCC | Cerebellum crus 1, lingual gyrus, cerebellum crus2, occipital pole, occipital fusiform gyrus, vermis 6 | decrease | | L/R | 502 | 0.000000 | -7.33 | 2 | -86 | -20 |
|  | Cerebellum crus 1, cerebellum crus 2 | decrease | | L | 137 | 0.000913 | -5.83 | -20 | -80 | -28 |
|  | Middle frontal gyrus | increase | | L | 111 | 0.002432 | 6.78 | -38 | 28 | 44 |
|  | Inferior temporal gyrus posterior division, temporal fusiform cortex posterior division | increase | | L | 57 | 0.049422 | 4.78 | -46 | -28 | -28 |
|  | **3 weeks > 6 weeks** |  | |  |  |  |  |  |  |  |
| MPFC | Paracingulate gyrus, cingulate gyrus anterior division | increase | | L/R | 89 | 0.022831 | 4.65 | -2 | 42 | 26 |
|  | Lateral occipital cortex, angular gyrus | decrease | | R | 70 | 0.036408 | -5.41 | 52 | -62 | 26 |
| LP/ R | Frontal pole | decrease | | L/R | 781 | 0.000000 | -6.05 | 26 | 60 | 4 |
|  | Angular gyrus | decrease | | R | 86 | 0.001169 | -5.17 | 54 | -48 | 30 |
| PCC | Superior temporal gyrus, planum polare, central opercular cortex, middle temporal gyrus, Heschl’s gyrus | decrease | | R | 248 | 0.000009 | -5.23 | 56 | -2 | -2 |
|  | Vermis 9, cerebellum crus1, cerebellum 6, cerebellum 8, cerebellum 9, cerebellum crus 2, cerebellum 7b | | increase | L | 244 | 0.000009 | 5.38 | -16 | -66 | -30 |
|  | Cerebellum crus1, cerebellum 6, occipital fusiform gyrus, cerebellum crus 2, lingual gyrus, cerebellum 8 | | increase | R | 243 | 0.000009 | 5.83 | 16 | -78 | -24 |
|  | Planum polare, superior temporal gyrus, insular cortex, middle temporal gyrus | | decrease | L | 105 | 0.003108 | -5.11 | -46 | -8 | -16 |
|  | **6 weeks > 9 weeks** | |  |  |  |  |  |  |  |  |
| LP/ L | Lingual gyrus, vermis 4 5 , cerebellum 6, vermis 6, cerebellum 4 5 | | increase | R | 453 | 0.000000 | 5.77 | 10 | -62 | -12 |
|  | Cerebellum crus 1, cerebellum crus 2 | | decrease | L | 132 | 0.00062 | -5.82 | -46 | -64 | -30 |
|  |  | |  |  |  |  |  |  |  |  |
|  | **9 weeks > 12 weeks** | |  |  |  |  |  |  |  |  |
| PCC | Thalamus | | decrease | R | 114 | 0.002786 | -5.20 | 24 | -32 | 16 |
|  | **12 weeks > 24 weeks** | |  |  |  |  |  |  |  |  |
| MPFC | Cingulate gyrus anterior division, paracingulate gyrus | | increase | R | 85 | 0.010135 | 4.51 | 10 | 26 | 30 |
| LP/ L | Superior parietal lobule | | decrease | L | 67 | 0.030369 | -5.10 | -40 | -46 | 60 |
| PCC | Insular cortex, central opercular cortex, Heschl’s gyrus | | increase | R | 275 | 0.000003 | 4.94 | 44 | -14 | 10 |
|  | Insular cortex, Heschl’s central opercular cortex, planum polare | | increase | L | 250 | 0.000004 | 5.92 | -42 | -12 | 6 |
|  |  | |  |  |  |  |  |  |  |  |

Note. DMN (Default Mode Network); L (left); R (right); MPFC (medial prefrontal cortex); PCC (posterior cingulate cortex); LP/ L (left lateral parietal lobules); LP/ R (right lateral parietal lobules)

**Table S5.** Functional connectivity maps of the FPN in postpartum men throughout the postpartum period (t-contrasts from random-effects GLM, p < .05, cluster-size p-FDR corrected; voxel threshold: p < .001 p-uncorrected).

| **Network** | **Anatomical Region** | **Connectivity** | **Side** | **Size** | **p- value** | **T** | **x** | **y** | **z** |
| --- | --- | --- | --- | --- | --- | --- | --- | --- | --- |
|  | **Childbirth > 3 weeks** |  |  |  |  |  |  |  |  |
| LPFC/ R | Middle temporal gyrus, inferior temporal gyrus | decrease | L | 109 | 0.002620 | -4.56 | -64 | -36 | -16 |
|  | Superior frontal gyrus, paracingulate gyrus | increase | R | 104 | 0.002620 | 4.94 | 12 | 32 | 34 |
|  | **3 weeks > 6 weeks** |  |  |  |  |  |  |  |  |
| LPFC/ R | Superior frontal gyrus | decrease | L | 51 | 0.030361 | -4.65 | -16 | 16 | 50 |
|  | **9 weeks > 12 weeks** |  |  |  |  |  |  |  |  |
| LPFC/L | Middle temporal gyrus posterior division, superior temporal gyrus posterior division | increase | R | 137 | 0.000248 | 5.07 | 58 | -16 | -6 |
|  | Cerebellum 9, cerebellum 8, vermis 10, cerebellum 10, | increase | L | 137 | 0.000248 | 5.43 | -10 | -42 | -44 |

Note. FPN (Frontoparietal Network); L (left); R (right); LPFC /L (left lateral prefrontal cortex); LPFC/ R (right lateral prefrontal cortex)

**Table S6.** Functional connectivity maps of from bivariate regression model showing significant correlations between PPAS Total scores and resting-state functional connectivity of the left and right amygdala in postpartum men throughout the postpartum period (t-contrasts from random-effects GLM, p < .05, cluster-size p-FDR corrected; voxel threshold: p < .001 p-uncorrected).

| **R / L AMY** | **Anatomical Region** | **Association** | **Side** | **Size** | **p-value** | **T** | **X** | **Y** | **z** |
| --- | --- | --- | --- | --- | --- | --- | --- | --- | --- |
|  | **3 weeks** |  |  |  |  |  |  |  |  |
| R | Cingulate gyrus posterior division, precuneus cortex | negative | R | 95 | 0,008522 | -5,22 | 8 | -36 | 44 |
| L | Precuneus cortex, Intracalcerine cortex, lingual gyrus, supracalcerine cortex | negative | L/R | 674 | 0,000000 | -5,53 | 8 | -70 | 10 |
|  | Precentral gyrus, postcentral gyrus | positive | L | 115 | 0,000667 | 5,01 | -10 | -30 | 52 |
|  | Cingulate gyrus | positive | L/R | 88 | 0,002334 | 5,61 | 00 | 16 | 14 |
|  | Precentral gyrus | negative | L | 56 | 0,015927 | -5,41 | -48 | 2 | 38 |
|  |  |  |  |  |  |  |  |  |  |
|  | **6 weeks** |  |  |  |  |  |  |  |  |
| R | Hippocampus | positive | L | 124 | 0,001388 | 4,95 | -28 | -36 | -2 |
|  | Parahippocampal gyrus | positive | R | 58 | 0,038065 | 6,06 | 24 | -6 | -32 |
|  | **9 weeks** |  |  |  |  |  |  |  |  |
| R | Precuneus cortex, postcentral gyrus | negative | R | 67 | 0,015188 | -4,99 | 14 | -34 | 50 |
| L | Thalamus | positive | L/R | 113 | 0,001768 | 6,12 | 2 | -18 | 16 |
|  | Precuneus | negative | L/R | 79 | 0,006778 | -5,61 | 2 | -56 | 48 |
|  | **12 weeks** |  |  |  |  |  |  |  |  |
| R | Insular cortex, frontal operculum cortex | negative | R | 294 | 0,000001 | -8,32 | 28 | 22 | 12 |
|  | Superior temporal gyrus anterior division, middle temporal gyrus anterior division, planum polare, Heschl's gyrus | negative | L | 178 | 0,000037 | -5,4 | -58 | -8 | -8 |
|  | Heschl's gyrus, middle temporal gyrus posterior division, superior temporal gyrus posterior division, central opercular cortex, planum polare | negative | R | 115 | 0,000774 | -4,71 | 54 | -16 | 6 |
| L | Parahippocampal gyrus posterior division, cerebellum 4 5, cerebellum 3, hippocampus | positive | L | 161 | 0,000157 | 5,92 | -16 | -32 | -12 |
|  | Heschl's gyrus, central opercular cortex, planum polare | negative | R | 100 | 0,002514 | -5,3 | 44 | -20 | 8 |
|  | Cingulate gyrus anterior division | negative | R | 53 | 0,036079 | -4,78 | 6 | 24 | 24 |
|  | Superior frontal gyrus, juxtapositional lobule cortex- supplementary motor cortex | negative | R | 51 | 0,036079 | -4,94 | 8 | 10 | 56 |
|  | Parahippocampal gyrus anterior division | positive | L | 54 | 0,046381 | 5,43 | -18 | -14 | -34 |

Note. AMY (amygdala); L (left); R (right); PPAS (Paternal Attachment Scale)

**Table S7.** Functional connectivity maps of from bivariate regression model showing significant correlations between PPAS subscale quality of attachment scores and resting-state functional connectivity of the left and right amygdala in postpartum men throughout the postpartum period (t-contrasts from random-effects GLM, p < .05, cluster-size p-FDR corrected; voxel threshold: p < .001 p-uncorrected).

| **R / L AMY** | **Anatomical Region** | **Association** | **Side** | **Size** | **p-value** | **T** | **X** | **Y** | **z** |
| --- | --- | --- | --- | --- | --- | --- | --- | --- | --- |
|  | **3 weeks** |  |  |  |  |  |  |  |  |
| L | Thalamus | positive | L | 148 | 0,000241 | 5,27 | 8 | -12 | 2 |
|  | **6 weeks** |  |  |  |  |  |  |  |  |
| R | Angular gyrus, supramarginal gyrus posterior division, parietal operculum cortex | negative | L | 255 | 0,000004 | -6,17 | 58 | -46 | 18 |
|  | Juxtapositional lobule cortex- supplementary motor cortex | negative | R | 108 | 0,002002 | -5,05 | -26 | 6 | 36 |
|  | Inferior temporal gyrus posterior division | positive | L | 96 | 0,00269 | 6,15 | -46 | -32 | -20 |
| L | Supracalcerine cortex | positive | L | 103 | 0,009137 | 5,24 | -24 | -66 | 10 |
|  | **9 weeks** |  |  |  |  |  |  |  |  |
| R | Thalamus | positive | L | 54 | 0,016542 | 4,97 | -6 | -18 | 6 |
| L | Precentral gyrus | positive | R | 80 | 0,009193 | 5,32 | 52 | 2 | 42 |
|  | Paracingulate gyrus | negative | L | 52 | 0,033917 | -4,87 | -10 | 28 | 40 |
|  | **12 weeks** |  |  |  |  |  |  |  |  |
|  | Superior temporal gyrus anterior division, middle temporal gyrus anterior division, temporal pole, planum polare | negative | L | 494 | 0 | -6,5 | -60 | -10 | -8 |
| R | Insular cortex, frontal operculum cortex | negative | R | 316 | 0 | -8,03 | 28 | 22 | 12 |
|  | Middle temporal gyrus posterior division, superior temporal gyrus posterior division, planum polare | negative | R | 176 | 0,000039 | -5 | 50 | -14 | -16 |
|  | Central operculum cortex, frontal operculum cortex, inferior frontal gyrus | negative | L | 96 | 0,001822 | -4,67 | -40 | 6 | 12 |
|  | Heschl's gyrus, planum polare | negative | R | 76 | 0,005356 | -4,59 | 46 | -16 | 9 |
|  | Planum polare, Heschl's gyrus, insular cortex, inferior temporal gyrus posterior division, central opercular cortex, temporal fusiform cortex posterior division | negative | R | 453 | 0 | -6,65 | 42 | -22 | 4 |
|  | Parahippocampal gyrus posterior division, cerebellum 4 5, cerebellum 3 | positive | L | 136 | 0,00023 | 5,47 | -18 | -34 | -16 |
| L | Parahippocampal gyrus anterior division | positive | L | 90 | 0,00238 | 5,44 | -18 | -18 | -34 |

Note. AMY (amygdala); L (left); R (right); PPAS (Paternal Attachment Scale)

**Table S8.** Functional connectivity maps of from bivariate regression model showing significant correlations between PPAS subscale absence of hostility scores and resting-state functional connectivity of the left and right amygdala in postpartum men throughout the postpartum period (t-contrasts from random-effects GLM, p < .05, cluster-size p-FDR corrected; voxel threshold: p < .001 p-uncorrected).

| **R / L AMY** | **Anatomical Region** | **Association** | **Side** | **Size** | **p-value** | **T** | **X** | **Y** | **z** |
| --- | --- | --- | --- | --- | --- | --- | --- | --- | --- |
|  | **3 weeks** |  |  |  |  |  |  |  |  |
| R | Postcentral gyrus, cingulate gyrus posterior division, precentral gyrus | negative | R | 285 | 0,000002 | -6,36 | 10 | -32 | 46 |
|  | Middle frontal gyrus, frontal pole, superior frontal gyrus | negative | L | 142 | 0,00046 | -4,92 | -34 | 34 | 36 |
|  | **9 weeks** |  |  |  |  |  |  |  |  |
| R | Inferior frontal gyrus, frontal operculum cortex | negative | L | 151 | 0,000164 | -5,43 | -44 | 32 | 8 |
| L | Frontal pole | negative | R | 110 | 0,001429 | -6,4 | 42 | 54 | -10 |
|  |  |  |  |  |  |  |  |  |  |
|  | **12 weeks** |  |  |  |  |  |  |  |  |
| R | Frontal operculum cortex | negative | R | 273 | 0,000001 | -8,19 | 26 | 32 | 12 |
| L | Cingulate gyrus anterior division | negative | R | 43 | 0,046104 | -4,92 | 9 | 26 | 18 |

Note. AMY (amygdala); L (left); R (right); PPAS (Paternal Attachment Scale)

**Table S9.** Functional connectivity maps of from bivariate regression model showing significant correlations between PPAS subscale pleasure in interaction scores and resting-state functional connectivity of the left and right amygdala in postpartum men throughout the postpartum period (t-contrasts from random-effects GLM, p < .05, cluster-size p-FDR corrected; voxel threshold: p < .001 p-uncorrected).

| **R / L AMY** | **Anatomical Region** | **Association** | **Side** | **Size** | **p-value** | **T** | **X** | **Y** | | **z** | |
| --- | --- | --- | --- | --- | --- | --- | --- | --- | --- | --- | --- |
|  | **3 weeks** |  |  |  |  |  |  |  |  | |  |
| R | Cerebellum 10, cerebellum 9, cerebellum 8 | positive | R | 232 | 0,000016 | 5,38 | 20 | -40 | -40 | |  |
|  | **9 weeks** |  |  |  |  |  |  |  |  | |  |
| R | Middle frontal gyrus | negative | R | 209 | 0,00001 | -5,87 | 32 | 32 | 40 | |  |
|  | **12 weeks** |  |  |  |  |  |  |  |  | |  |
| L | Central opercular cortex, Heschl's gyrus, planum temporale, superior temporal gyrus anterior division | negative | R | 148 | 0,000006 | -4,96 | 60 | -6 | 6 | |  |

Note. AMY (amygdala); L (left); R (right); PPAS (Paternal Attachment Scale)
